# IpiRId: Integrative approach for piRNA prediction using genomic and epigenomic data

**DOI:** 10.1371/journal.pone.0179787

**Published:** 2017-06-16

**Authors:** Anouar Boucheham, Vivien Sommard, Farida Zehraoui, Adnane Boualem, Mohamed Batouche, Abdelhafid Bendahmane, David Israeli, Fariza Tahi

**Affiliations:** 1 IBISC, Univ Evry, Université Paris-Saclay, Evry, France; 2 Institute of Plant Sciences Paris-Saclay, IPS2, INRA, CNRS, University of Paris-Sud, University of Evry, University of Paris-Diderot, Sorbone Paris-Cité, University of Paris-Saclay, Orsay, France; 3 Faculty of ISCA, Constantine University 3, Constantine, Algeria; 4 College of NTIC, Constantine University 2, Constantine, Algeria; 5 Genethon, Inserm U951 INTEGRARE, Univ Evry / Paris-Saclay, Evry, France; Harbin Institute of Technology Shenzhen Graduate School, CHINA

## Abstract

Many computational tools have been proposed during the two last decades for predicting piRNAs, which are molecules with important role in post-transcriptional gene regulation. However, these tools are mostly based on only one feature that is generally related to the sequence. Discoveries in the domain of piRNAs are still in their beginning stages, and recent publications have shown many new properties. Here, we propose an integrative approach for piRNA prediction in which several types of genomic and epigenomic properties that can be used to characterize these molecules are examined. We reviewed and extracted a large number of piRNA features from the literature that have been observed experimentally in several species. These features are represented by different kernels, in a Multiple Kernel Learning based approach, implemented within an object-oriented framework. The obtained tool, called IpiRId, shows prediction results that attain more than 90% of accuracy on different tested species (human, mouse and fly), outperforming all existing tools. Besides, our method makes it possible to study the validity of each given feature in a given species. Finally, the developed tool is modular and easily extensible, and can be adapted for predicting other types of ncRNAs. The IpiRId software and the user-friendly web-based server of our tool are now freely available to academic users at: https://evryrna.ibisc.univ-evry.fr/evryrna/.

## Introduction

Non-coding RNAs (ncRNAs) play important roles in various cellular activities and are closely associated with cancer and other complex diseases, which has made their identification a critical issue in biological research [1, 2]. Different computational approaches for predicting ncRNAs have been proposed, based on homology information or on common features characterizing these molecules [3, 4], and most of these methods are developed for specific classes of ncRNAs. For example, a large number of tools have been developed for microRNAs, a widely studied class of ncRNAs. Among these tools we can cite for instance miRNAFold [3], miRBoost [4], miRNA-dis [5] and iMiRNA-PseDPC [6].

PIWI-interacting RNAs (piRNAs) are a novel class of endogenous small ncRNAs abundant in mammalian germline cells and interacting with the Piwi subfamily of proteins [7]. They play a vital role in the regulation of gene expression and are involved in the formation of germline cells via the “Ping-Pong” pathway. Nowadays, the main role attributed to piRNAs is the silencing of the mobile elements (retrotransposons and other repeat elements) in germ cells [8, 9]. They are the largest and most heterogeneous class of the small ncRNA family, thereby lacking clear secondary structure motifs and conservation in and between species, which makes their identification a challenging task.

With the development of a new generation of sequencing technologies (NGS), biologists can access huge volumes of sequencing data (e.g. RNA-seq data). Exploiting this amount of data requires computational tools for the identification of potential piRNAs, that could be validated by experimental techniques. Several computational tools have been proposed in the literature [10–12], almost of them based on machine learning techniques.

Several features of piRNAs have been discovered, many of them recently, suggesting that others will certainly be discovered in the next few years. The majority of these features are linked to the sequence. Recently, a category of tools has been introduced that aim to annotate and formulate RNA sequences with discrete vectors by focusing on their different features and properties, such as Pse-in-one [13] and repRNA [14]. Thus, almost all of the existing tools for piRNA prediction are based on these classical features, and are mostly based on only one kind of features [10–12].

In this paper, we present an integrative approach for predicting piRNAs, by considering many recently discovered features. For this purpose, we did a thorough study on what can characterize a piRNA from both genomic and epigenomic standpoints. Indeed, a piRNA can be characterized by its (i) sequence but also its (ii) positions on the chromatin, (iii) positions regarding sequence and/or structural motifs that can occur at the 5’ and/or the 3’ ends, (iv) possible occurrence in clusters, and (v) interaction with specific target sequences.

We then developed a generic tool, called IpiRId, based on the Multiple Kernel Learning (MKL) method [15]. This method, which combines several kernels representing different types of features, deals with the heterogeneity of the considered features. We define a set of generic kernels that could be directly used by instantiation according to the different types of piRNA’s features. Thanks to the proposed object-oriented framework, our tool is modular and easily extensible and modifiable, and enables testing each kernel separately, in order to perceive the feature conservation across species. The current version of our tool, which implements twelve different kernels, shows the outperformance and the advantages of an integrative analysis in piRNA prediction, when compared to all other existing tools, i.e. piRNApredictor [10], Piano [11], Pibomd [12] and piRPred [16]. IpiRId gives more than 90% in accuracy for each of three studied species: human, mouse and fly. More importantly, the prediction results are homogeneous for any species, which is not the case for the other tools.

The paper is organized as follows: we first present the exhaustive study we carried out on the different kinds of features that could be considered for predicting piRNAs. Then we explain how these features are implemented in an MKL approach that we implemented in an object-oriented framework. In the results section, we review the existing tools for piRNA prediction and show the results obtained by each of them, as well as the ones obtained by our tool IpiRId, on three considered species. And finally, we show the pertinence of reviewed piRNA features across species, before concluding.

## Materials and methods

### piRNA’s features in diverse organisms

**Fig 1:**
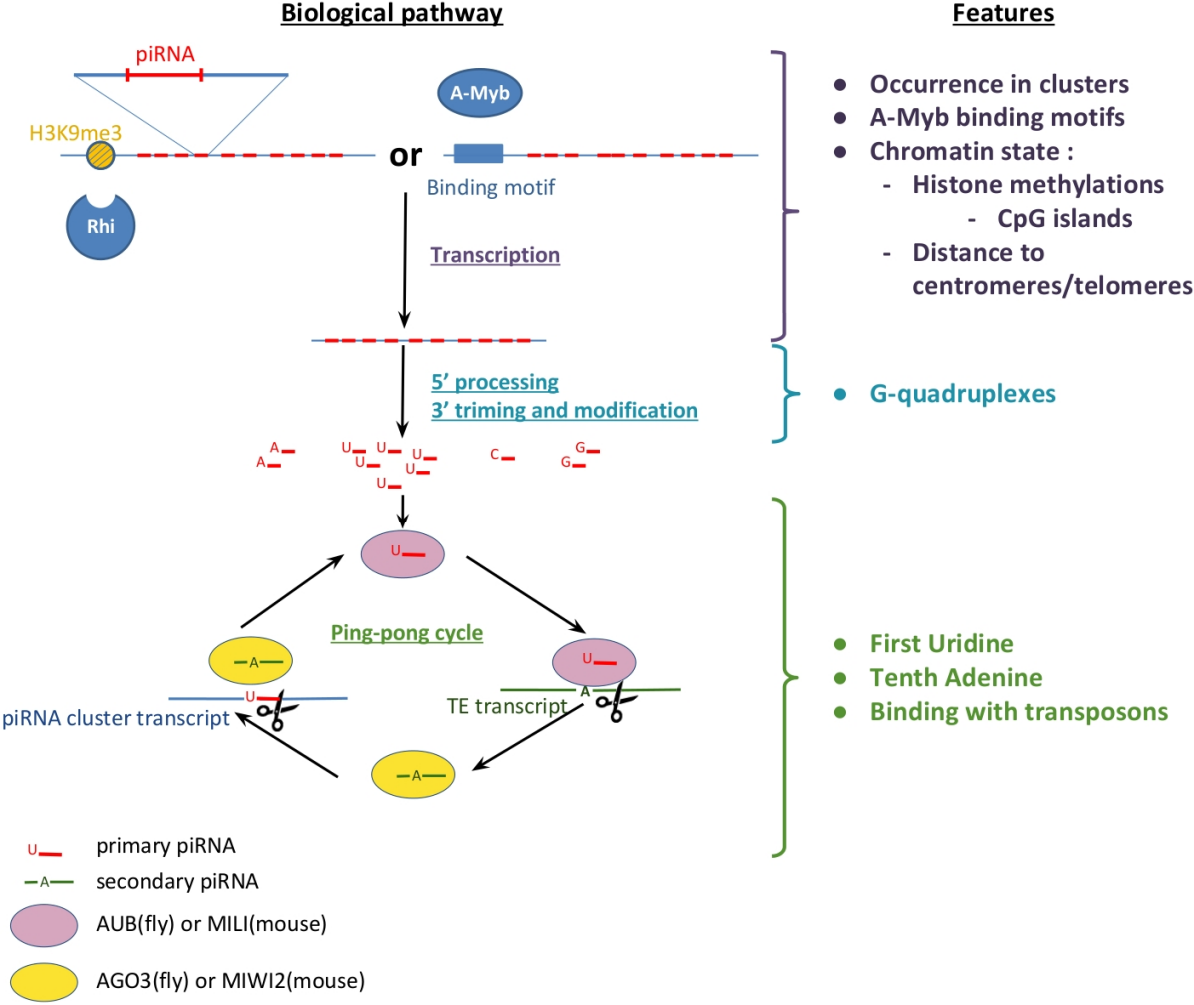
Relationship between piRNA biogenesis (transcription, processing and function) and measured features. (i) piRNA clusters can be transcribed if particular methylated histone (fly) or A-Myb promoter (mouse) is nearby; (ii) G-quadruplexes could have a role in piRNA processing and (iii) both first and tenth piRNA bases (respectively U and A) represent an important binding zone for Argonaute proteins, participating in a ping-pong cycle where the piRNA sequences bind with transposons.

We reviewed the recent studies on piRNA biogenesis and function and on other biological observations related to this molecule in diverse species, in order to deduce interesting features. In the following, we briefly summarize and categorize these features which are mainly related to: the function, the transcription, and other observed features, as shown in Fig 1.

#### Features related to the function

Recent studies revealed that both first (5’ nucleotide) and tenth piRNA bases represent an important binding zone for many Argonaute proteins [17]. Accordingly, PIWI and AUB proteins show a strong preference for 5’ uridine, while Ago3-associated piRNAs do not appear any enrichment for 5’ ‘U’ but tend to contain an adenosine as their tenth nucleotide, also called ping-pong signature [7, 18]. Another important piRNA characteristic concerns the principal role of this type of small ncRNA. piRNAs have been found to be antisense to transposable elements (TEs) which protects the genome from invasive TEs and maintains its integrity [19].

#### Features related to the transcription

During recent years, piRNAs have been shown to appear in clusters in mammals and insects species [18]. To better understand the transcription process, an important step is to take into account the state of the chromatin around the sequences and consider almost all epigenetic modifications. A recent study reports that most of the piRNA clusters in *Drosophila melanogaster* have been identified in pericentromeric and telomeric heterochromatin regions [20]. Furthermore, another study on the same species reports that piRNA clusters are often coated with H3 trimethylated on their lysine 9 (H3K9me3). Also, the transcription of some piRNA clusters requires Rhino which is a Heterochromatin Protein 1 (HP1) homolog and has a chromodomain (CD) which binds to H3K9me3 or H3K27me3 [21]. An alternative way to consider epigenetic modification is to rely on the genomic sequence by predicting CpG islands which have been shown to be linked to histone methylation [22]. Interestingly, further studies have investigated the transcription of piRNA clusters in *Mus musculus* and found that the transcription factor A-Myb is required for the expression of pachytene piRNAs. It was observed that A-Myb binds DNA near the transcription start site of pachytene piRNA clusters [23].

#### Other observed features

Several clusters of piRNAs have been studied in *Mus musculus* and some are bracketed by inverted repeats, allowing the formation of precursors containing double-stranded RNA [24]. In the same study, it was also found that some piRNA clusters are flanked by TEs such as SINE, LINE and LTR. This has been reported also in [25] where it was shown that the transposition of these elements can be also into piRNA clusters.

Finally, a recent study reports the presence of G-quadruplex motifs in mammal piRNA clusters and these structures may have a role in piRNA processing [26].

Table 1 summarizes the studied piRNA biological features, with for each, the species where it has been observed and/or validated.

**Table 1:** piRNA’s biological features over species.

Feature	Species	References
First Uridine	Fly, Mouse, Human, Rat, Nematode (C. elegans), Zebrafish and Silkworm (Bombyx mori)	[17–19]
Tenth Adenine	Human, Fly, Mouse, Zebrafish and Silkworm (Bombyx mori)	[17, 18]
Occurrence in clusters	Mammals and Insects	[18]
Binding with transposons	Mammals and Insects	[19]
CpG islands	Mammals	[22, 27]
G-Quadruplex	Human, Mouse, Rat and Macaque	[26]
Transposable elements presence	Mouse and Marmoset	[24, 25]
Promoter A-Myb	Mouse	[23]
Inverted repeats	Mouse	[24]
Distance to centromeres/telomeres	Fly	[7]
Histone methylation	Fly	[21]

### MKL methodology

We propose here an integrative approach for piRNA prediction based on supervised machine learning that considers different kinds of features.

Standard machine learning approach deals with features represented by vectors. To represent structured complex data, we use kernels. Since we have many heterogeneous features coming from different sources, we propose to use Multiple Kernel Learning (MKL) approach [28]. Each data source is represented by a kernel. This allow to obtain an homogeneous representation of the heterogeneous features. These kernels are then combined and the weight of each kernel (source) is tuned automatically inside the MKL algorithm. We thus build several kernels representing heterogeneous features and combine them in order to perform binary classification using a Support Vector Machine (SVM) classifier. We use the SPG-GMKL software [15] which employs spectral projected gradient descent-based optimizer in order to find the optimal combination of kernels. In this work, we choose a Gaussian kernel which is a universal kernel for feature representation. It consists of a square similarity matrix of size *N* * *N*, *N* being the size of the training dataset (positive and negative samples). Let *x* and *y* be two feature vectors or matrices representing two sequences. In this type of kernel, the inner product of *x* and *y* in the feature space is given by the following equation:

$$\begin{array}{c} k\left(x,y\right)=exp^{-\gamma ||x-y||2} \end{array}$$ (1)

The distance between *x* and *y* will be the Euclidian distance if *x* and *y* are vectors, and the Frobenius distance if *x* and *y* are matrices.

To fix an appropriate kernel parameter *γ*, we first use the Jaakkola’s heuristic [29] to calculate the initial value of *γ* as follows:

$$\begin{array}{c} \gamma JAAK=1/(2median{\left(distMat\right)}^{2}) \end{array}$$ (2)

Then we look for possible solution:

$$\begin{array}{c} \gamma =exp(i)*\gamma JAAK \end{array}$$ (3)

with *i* an integer in [−4, 4]. Each value of the parameter *γ* is evaluated by calculating the inter-cluster distance between positive and negative sequences. Finally, we choose the one giving the highest distance, which will lead to a better classification [30]. The inter-cluster distance is calculated as follows [30]:

$$\begin{array}{c} \delta \left(X_{+},X_{-}\right)=\frac{1}{l_{+}+l_{-}}\left(\sum_{x_{+}\in X_{+}}d\left(x_{+},{\bar{X}}_{-}\right)+\sum_{x_{-}\in X_{-}}d\left(x_{-},{\bar{X}}_{+}\right)\right) \end{array}$$ (4)

where *X*_+_ and *X*_−_ are positive and negative sequences, *l*_+_ and *l*_−_ are their corresponding sizes, and ${\bar{X}}_{-}$ and ${\bar{X}}_{+}$ are the class means of *X*_+_ and *X*_−_.

### Principal kernel classes and our object-oriented framework

**Fig 2:**
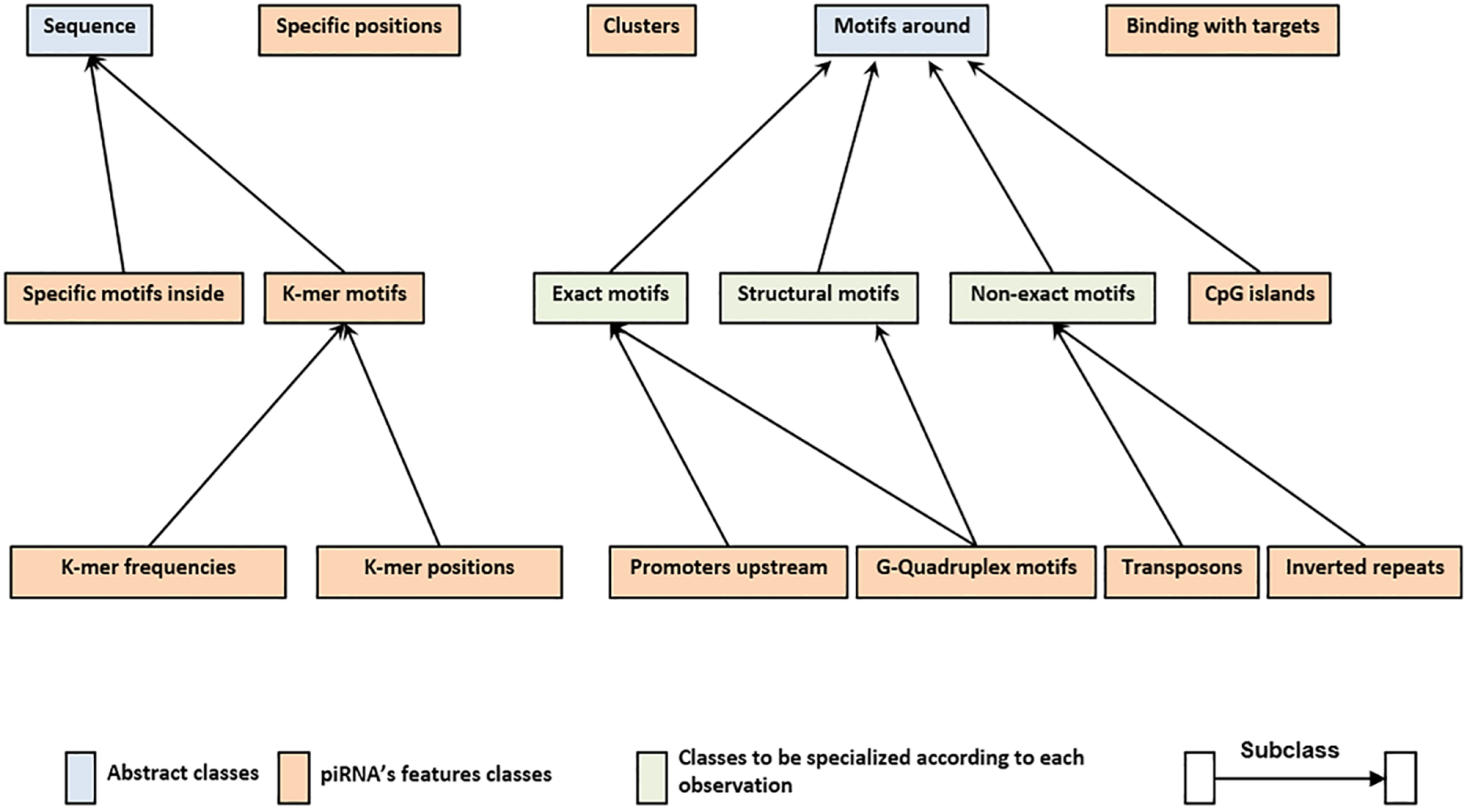
The different kernel classes defined in IpiRId and their hierarchical organisation.

We developed an object-oriented framework, called IpiRId, implemented in Java, which consists of different classes and sub-classes representing different kernels. Fig 2 gives the general architecture of our framework and the different classes we defined. Some classes are abstract (blue), since they do not correspond to implemented kernels, but their definition allows us to build a better hierarchical structuration. Besides, some classes of kernels can be directly instantiated for piRNA’s features (brown), and others must be specialized, according to each specific observation related to piRNAs (green).

In the following, we give a description of the principal classes of kernels for predicting piRNAs, and propose for each a methodology that allows considering in a computational manner features belonging to the kernel.

#### Specific motifs inside

This class of kernels represents the features corresponding to the presence/absence of motifs at specific positions in the sequence. Accordingly, we construct a *N*-dimensional binary vector containing the information about the presence or the absence of each motif, where *N* is the number of motifs.

#### K-mer motifs

K-mers are largely applied for sequence analysis, especially in the identification of ncRNAs, where many k-mer-based approaches have been proposed in the last years, as for piRNAs and microRNAs [10, 12, 31]. A common step in these methods is feature extraction in which many features or k-mers are generated or in some cases extracted from strings based on specific observations. Then feature selection techniques are applied to identify the most pertinent and non-redundant ones for a specific species. In order to achieve high predictive performance, we use a non-identified character ‘*X*’, which can be ‘A’, ‘T’, ‘C’ or ‘G’, with a maximum occurrence probability of 0.4. Accordingly, we generate 3 588 patterns that represent all possible k-mers, for k = 1 to 5. After that, we perform a supervised selection on this ensemble which represents an *NP*-hard combinatorial optimization problem where we try to identify the most informative subset of k-mers that can achieve good prediction. This can be formulated as a feature selection problem where each pattern is a feature and each sequence refers to a sample. To handle selection, we employ the modified particle swarm optimization feature selection method proposed by [32, 33] with a predefined number of selected features, to identify the most representative *N* k-mers among all generated patterns. Finally, each sequence is represented by a *N*-dimentional vector, *N* representing the number of selected k-mers.

#### K-mer frequencies

In this subclass of the K-mer motifs class, the discriminative information used to perform the selection of k-mers is their frequencies in the sequence. Subsequently, the *N*-dimensional vector will contain the frequencies of the *N* k-mers divided by the sequence length.

#### K-mer positions

In this other subclass of the K-mer motifs class, the considered discriminative information is the position of each k-mer in the sequence. If a k-mer is present many times we keep the closest position to the beginning of the sequence and if it is never present the corresponding value is zero.

#### Clusters

In order to take into consideration the location on the chromosome of piRNA clusters, we propose a kernel, which takes into account the neighbors of each sequence in the genome. The neighbors in our approach represent the closest sequences that are located on the same chromosome as the target sequence and contained in the training set. We propose to find the *k*-nearest neighbors of each sequence and then to construct a (*K*+1)*(*K*+1) matrix containing the distances between all the sequences (the target sequence and its k-nearest neighbors). Each matrix represents a density ‘context’ of a target sequence in the training set without using the labels of the neighbors. The value of *k* depends on the number of piRNAs contained in a cluster. This value is a parameter that can be changed by the user.

#### Binding with targets

The principal information measured and considered in this class is the extent of the binding between piRNA sequences and given targets. For measuring the sequence/target binding information, several tools can be used, like the RNAplex tool [34]. Binding information can be represented as follows: opening brackets (“(”) to indicate paired nucleotides and dots (“.”) to indicate unpaired nucleotides. In order to get benefits of this information, we make use of the triplet structure sequence elements used for example in [35, 36] to predict pre-miRNAs based on their structure. These triplet structures consist of combining the middle nucleotide (‘A’, ‘T’, ‘C’ or ‘G’) of each three adjacent nucleotides, given that there are 8 (2^3^) possible structure compositions for any three adjacent nucleotides, to form 32 (4x8) different triplet elements that contain both folding and piRNA sequence information. Then, we count the frequencies of each triplet element for each sequence. As a result, a 32-dimensional vector will represent its folding information.

#### Specific positions

This class of kernels takes into account the possible occurrence of the piRNA near/close to specific observations on the genome. To integrate this information in a computational manner, we measure the distance between the sequence and these observations. As each observation can have one or many positions on the genome, we need to establish selection criteria according to the biological sense of this observation to choose the best position to be considered (generally the nearest one). Moreover, a piRNA sequence can have many positions, thus the position with the lowest distance is conserved. Finally, we build a *N*-dimensional vector containing the best distances to the *N* observations.

#### Motifs around

Developing a generic implementation for any feature belonging to this class is a difficult task since the discriminant information to be investigated depends on the biological specificities of the considered feature. However, this class of kernels is based on the reference genome, in order to search for motifs upstream and/or downstream of the piRNA sequence. Also it considers the closest distance to the motif and the discovered motif length as discriminative information as well as other specific data. Several subclasses will therefore be built according to the type of motifs searched for, which will depend on each specific observation. In the following, we describe these different subclasses.

#### Promoters upstream

To consider the role of transcription factors in piRNA prediction, we make use of the identified binding motifs related to their promoters. In most cases there is not an explicit binding motif but rather several motifs that can share a consensus. Therefore, we use the reference genome to browse upstream of the positions of each occurrence of a given sequence on the genome. We start from the 5’ of the sequence and search upstream for all possible motifs and stop at the first one found. We maintain three types of information on the discovered motif: the motif length (*L*), the distance (*D*) between the motif and the sequence, and a probability calculated as 4^*L*^/*D*, which allows selecting the position with the closest motif to the sequence as well as the longest one.

#### Transposons

To study the presence of TEs around piRNAs, we used the RepeatMasker software (http://www.repeatmasker.org). We expect to find TEs around and in piRNA clusters. For that purpose, we look until *D* kb upstream and *D* kb downstream of the given sequence. Based on the ReapetMasker outputs, we calculate two kinds of information: the cumulated identity and the cumulated length for each TE. The identity is calculated as:

$$\begin{array}{c} Identity=1-RM-RD-RS \end{array}$$ (5)

where *RM*, *RD* and *RS* are, respectively, the ratio of mismatch, deletion and substitution. If the piRNA sequence has multiple positions, we choose the position with highest cumulated identity. Since the different TEs have not the same chance to be found around a piRNA (for example, LINE elements are indeed more often found in piRNA clusters than SINE elements and then LTR elements [25]); they are therefore weighted accordingly. Finally, each sequence is represented by a (2 * *N*)-dimensional vector, where *N* is the number of considered TEs.

#### Inverted repeats

To evaluate the presence of inverted repeats in the proximity of a given sequence, we use the method used in [24]. We make use of the genomic sequence *D* kb upstream and *D* kb downstream of the sequence and compare the obtained sequence to its complement with BLAST (bl2seq in gapless mode). Alignments longer than 20 bases and with more than 90% identity are considered. Accordingly, we calculate the mean of their length and the cumulated number of their identities. Each sequence is therefore represented by a 2-dimensional vector.

#### G-quadruplex

We look here at G-quadruplex structures in the vicinity of each sequence. To this purpose, we use a Python script (http://bioinformatics-misc.googlecode.com/svn-history/r16/trunk/quadparser.py), allowing to predict the G-quadruplexes *D* kb upstream and *D* kb downstream on the strand of the sequence and on the opposite strand. Then, we calculate five kinds of information: the distance to the nearest G-quadruplex on the strand of the sequence, the distance to the nearest G-quadruplex on the opposite strand, the number of occurrences of G-quadruplexes on both strands, and finally the cumulated length of all G-quadruplexes. Each sequence is therefore represented by a 5-dimensional vector. If a sequence has multiple positions, we choose the position with the nearest G-quadruplex on its strand.

#### CpG islands

We also consider the methylation differently by using only the genomic sequence upstream from the given piRNA and predict CpG islands on it. For that purpose, we use newcpgreport tool [37] to detect CpG islands with a minimum length of *L* nucleotides. For each sequence, the *D* Kb upstream genomic sequence is given to newcpgreport which calculates the related information: distance to the nearest CpG island, number of the predicted CpG islands, mean of the observed expressed ratio, mean of the lengths of the islands and mean of the sum of C+G bases in the islands. Each sequence is then represented by a 5-dimensional vector. If a sequence has multiple positions, we choose the one with the lowest distance to an upstream predicted CpG island.

To summarize, IpiRId is currently composed of twelve kernels which are listed in Table 2.

**Table 2:** IpiRId’s kernels instantiation. (*D*: distance; *L*: minimal length).

Kernel	Class	Instantiation parameters
U1|A10	Specific motifs inside	{motif,position}: {U,1}, {A,10}
K-merFreq	K-mer frequencies	*N* (number of k-mers) = 32 motifs
K-merPos	K-mer positions	*N* (number of k-mers) = 32 motifs
TE binding	Binding with targets	target: Transposable elements (TE)
CentroTelo	Specific positions	observation: centromer, telomeres
Histone	Specific positions	observation: H3K9me3, H3K27me3
Cluster	Clusters	*K* (number of neighbours) = 4
A-Myb	Promoters upstream	promoter: A-Myb; *D* = 40 kb
G-Quadruplex	G-quadruplex	*D* = 40 kb
CpG islands	CpG islands	*L* = 100; *D* = 20 kb
LINE|SINE|LTR	Transposons	TE: LINE, SINE, LTR; *D* = 40 kb
InvertRep	Inverted repeats	*D* = 40 kb

## Results and discussion

In this section, we present the study conducted in order to assess the advantages of our integrative approach for piRNA prediction. We show the cross-validation results of IpiRId tool compared to other tools from literature on three species, human, mouse and fly, and discuss the pertinence of each kernel, representing a feature, according to these species.

### Dataset construction

In order to build high-quality training and test datasets, we create three datasets with both positive and negative piRNA sequences, each of which refers to one of three species considered in this study: human (*H*omo sapiens), mouse (*M*us musculus) and fly (*D*rosophila melanogaster). Positive non-redundant piRNA sequences were collected from both piRNAbank [38] (http://pirnabank.ibab.ac.in/) and piRBase [39] (www.regulatoryrna.org/database/piRNA/) databases, from where we downloaded 32 208, 39 986 and 18 508 human, mouse and fly piRNA sequences, respectively. For negative sequences, we considered:

- 449, 244 and 93 human, mouse and fly tRNA sequences, respectively, downloaded from the genomic tRNA database (http://lowelab.ucsc.edu/GtRNAdb/).
- 1 747, 712 and 288 human, mouse and fly mature miRNA high-confidence sequences [40], respectively, downloaded from miRBase (http://www.mirbase.org/).
- 9 113, 4 896 and 740 human, mouse and fly exonic region sequences, respectively, with exact length between 25-33 for both human and mouse and 22-35 for fly, downloaded from Ensembl (www.ensembl.org/index.html).

All positive and negative sequences were aligned onto human hg38, mouse mm10 and fly dm6 reference genomes using Bowtie software, which is used by piRbase to determine genomic positions [39], without allowing any gaps and allowing a maximum of one mismatch for sequences that do not match exactly. Except for mature miRNAs which are included in precursors (pre-miRNAs), realigning them will produce too many positions. Thus, we use the positions provided by miRBase 21 and lift them to the appropriate reference genome using Liftover tool from the UCSC Genome Browser [41].

In order to build the TE binding kernel, transposons were gathered from “rmsk” table of the UCSC Genome Browser [41], excluding those with rich annotation, repeated nucleotides and redundant transposons. For computational reasons, we considered only transposons with length between 35 and 100 nt, and finally selected randomly 1 000 from the whole set. This allows us to look at the same number of transposons of similar length for each species. This length is a parameter that could be fixed by the user.

Finally, epigenetics ChIP-Seq data represented by the positions of histones H3K9me3 and H3K27me3 were taken from the NCBI epigenomic repository. The considered tissues/cells in our study are: T cells for human (Downloaded from: http://dir.nhlbi.nih.gov/papers/lmi/epigenomes/hgtcell.aspx), embryonic stem cells for mouse (Downloaded from: http://www.ncbi.nlm.nih.gov/epigenomics/166 for H3K9me3 and http://www.ncbi.nlm.nih.gov/epigenomics/164 for H3K27me3) and ovaries (for H3K9me3) and testis (for H3K27me3) for fly (Downloaded from: http://www.ncbi.nlm.nih.gov/geo/query/acc.cgi?acc=GSM1121659 for H3K9me3 and http://www.ncbi.nlm.nih.gov/geo/query/acc.cgi?acc=GSM480447 for H3K27me3). We used also Liftover tool [41] to lift from the downloaded epigenetics data assemblies to the appropriate ones adopted for each considered species.

Table 3 summarizes the different downloaded data sets used in our experiments.

**Table 3:** The downloaded data used in our integrative approach for piRNAs identificatiton across species.

Species/Dataset	positive	negative			chip-seq data		transposons	reference genome assembly
	piRNA	tRNA	miRNA	exonic regions	H3K9me3	H3K27me3		
Homo sapiens	32 208	449	1 747	9 113	6 346 007	8 968 536	903 140	hg38
Mus Musculus	39 986	244	712	4 896	2 751	1 232 402	3 504 253	mm10
Drosophila melanogaster	18 508	93	288	740	508	2 322	803 255	dm6

### piRNA prediction tools comparison

Few tools have been proposed for predicting piRNAs. The first published tool is piRNApredictor which is based on k-mer motif frequencies [10]. It uses the Fisher method to select the most discriminate k-mers (k = 1-5) and then performs an improved Fisher with a threshold to classify the sequences. Another k-mer based tool, called Pibomd, was recently proposed [12]. It searches for all 5-mer and 4-mer motifs with three common nucleotides and belonging to 40% of the training sequences. The frequencies of all k-mers found are then used in an SVM to classify the sequences. Another recently proposed tool, called Piano, is based on piRNA/transposon binding information [11]. It uses SeqMap tool to select the sequences with three mismatches or less using the option “/*outputallmatches*”. Then, it uses RNAplex to fold each sequence with transposons with a maximum of three mismatches, in order to perform prediction using SVM classifier. In our team, we recently proposed a tool for piRNA prediction called piRPred based on the MKL approach [16], composed of three kernels implementing respectively the following features: (i) presence of uridine (‘U’) at the first position of the sequence and k-mer motif frequency (k-mers considered by [10] as the most discriminant ones), (ii) occurrence into clusters, and (iii) distance to centromeric and telomeric regions.

To undertake a comparison between our tool IpiRId and the other existing tools, we have retrained these tools on our datasets using 5-cross validation technique. We should note that we had many problems to retrain piRNApredictor, Piano and Pibomd tools, since they are functional only in prediction mode, and information to retrain them are not mentioned in their manuals or publications.

As described above, three species were considered in our study: human, mouse and fly. We built a dataset containing 5000 piRNA sequences and 5000 pseudo piRNA sequences for both human and mouse species and 1100 piRNA sequences and 1100 pseudo piRNA sequences for fly. These sequences were obtained by random selection from the original downloaded set of data for each species.

Table 4 reports the 5-fold cross validation results of our tool IpiRId and the other existing tools (piRNApredictor, Piano, Pibomd and piRPred) on the three species. The results are given according to five measures, usually used in supervised classification tasks: Sensitivity (Se), Specificity (Sp), Precision (Pre), Accuracy (Acc) and F1 score (F1). They are described below using the following abbreviations: *TP*: True Positive, *FP*: False Positive, *TN*: True Negative and *FN*: False Negative.

$$\begin{array}{c} Accuracy\left(Acc\right)=\frac{TP+TN}{TP+TN+FP+FN}*100 \end{array}$$ (6)

$$\begin{array}{c} Sensitivity\left(Se\right)=\frac{TP}{TP+FN}*100 \end{array}$$ (7)

$$\begin{array}{c} Specificity\left(Sp\right)=\frac{TN}{TN+FP}*100 \end{array}$$ (8)

$$\begin{array}{c} Precision\left(Pre\right)=\frac{TP}{TP+FP}*100 \end{array}$$ (9)

$$\begin{array}{c} F1\;score\left(F1\right)=\frac{2TP}{2TP+FP+FN}*100 \end{array}$$ (10)

**Table 4:** Performance comparison. 5-fold cross-validation results of IpiRId and other existing tools according to: Accuracy (Acc), Sensitivity (Se), Specificity (Sp), Precision (Pre) and F1 score (F1).

Tool/Species	Human					Mouse					Fly				
	Acc	Se	Sp	Pre	F1	Acc	Se	Sp	Pre	F1	Acc	Se	Sp	Pre	F1
piRNApredictor	71.85+-1.53	48.40	**95.5**	**91.49**	63.30	70.95+-1.15	47.79	94.10	89.01	62.19	52.17+-3.72	63.90	40.45	51.76	57.19
Piano	50	0	100	0	0	50	0	100	0	0	87.9+-1.472	78.90	96.90	96.22	86.70
Pibomd	78.13+-1.38	78.05	78.21	78.17	78.11	79.13+-1.19	79.43	78.82	78.94	79.18	66.08+-4.02	70.44	61.72	64.78	67.94
piRPred	81.20+-1.25	80.54	81.86	81.67	81.07	90.92 +-0.51	90.36	91.48	91.39	90.87	86.36+-2.33	86	86.72	86.66	86.30
IpiRId	**90.09+-0.25**	**90.56**	89.62	89.73	**90.13**	**93.66+-0.46**	**90.74**	**96.58**	**96.37**	**93.47**	**92.59+-1.87**	**87.27**	**97.90**	**97.67**	**92.12**

The results clearly show the outperformance of our tool. IpiRId gives more than 90% of accuracy in all species, as well as a close sensitivity, specificity, precision and F1 score that are all around 90%. Pibomd, the tool showing the third best results (after piRPred, a previous tool developed by our team), gives an accuracy, as well as a sensitivity, specificity, precision and F1 score less than 80% in all species (less than 70% in fly). Note that Piano works only in *Drosophila melanogaster*. This could be due for selecting only sequences matching with transposons and for each sequence selecting only the matching transposons. Thus, Piano doesn’t consider the same subset of transposons for each sequence to calculate and doesn’t consider the matching transposons with a gap. Especially that it has been observed that only 17% of piRNAs map to transposons in mammals [18, 42].

**Fig 3:**
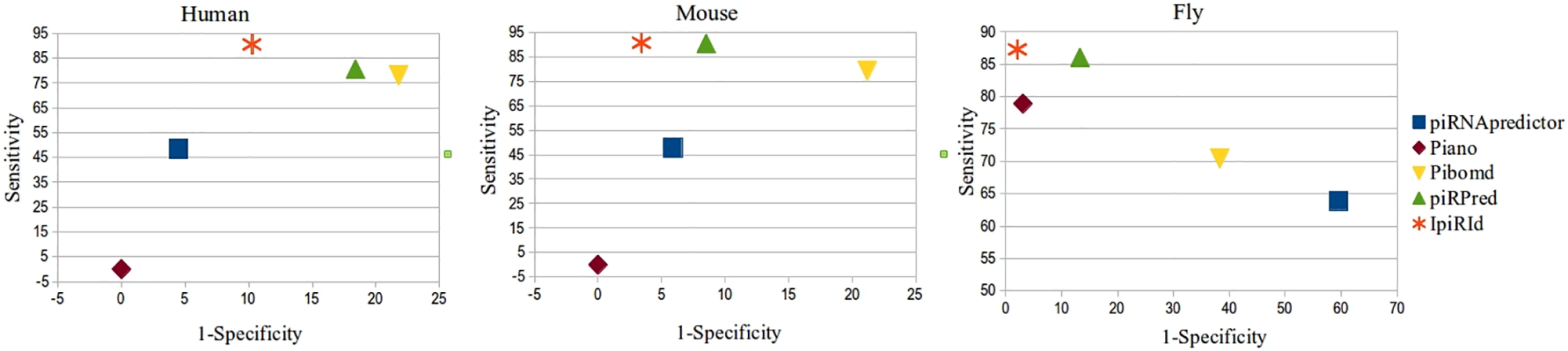
ROC space and plots of the 5-fold cross-validation results of IpiRId and other tools across species, with fixed parameters.

The ROC spaces given in Fig 3 and corresponding to the 5-fold cross-validation results obtained by IpiRId, piRPred, Pibomd, Piano and piRNApredictor show clearly that IpiRId gives the best compromise between specificity and sensitivity for all considered species, particularly for Mouse and Fly. The other tools give very heterogeneous results across species.

**Fig 4:**
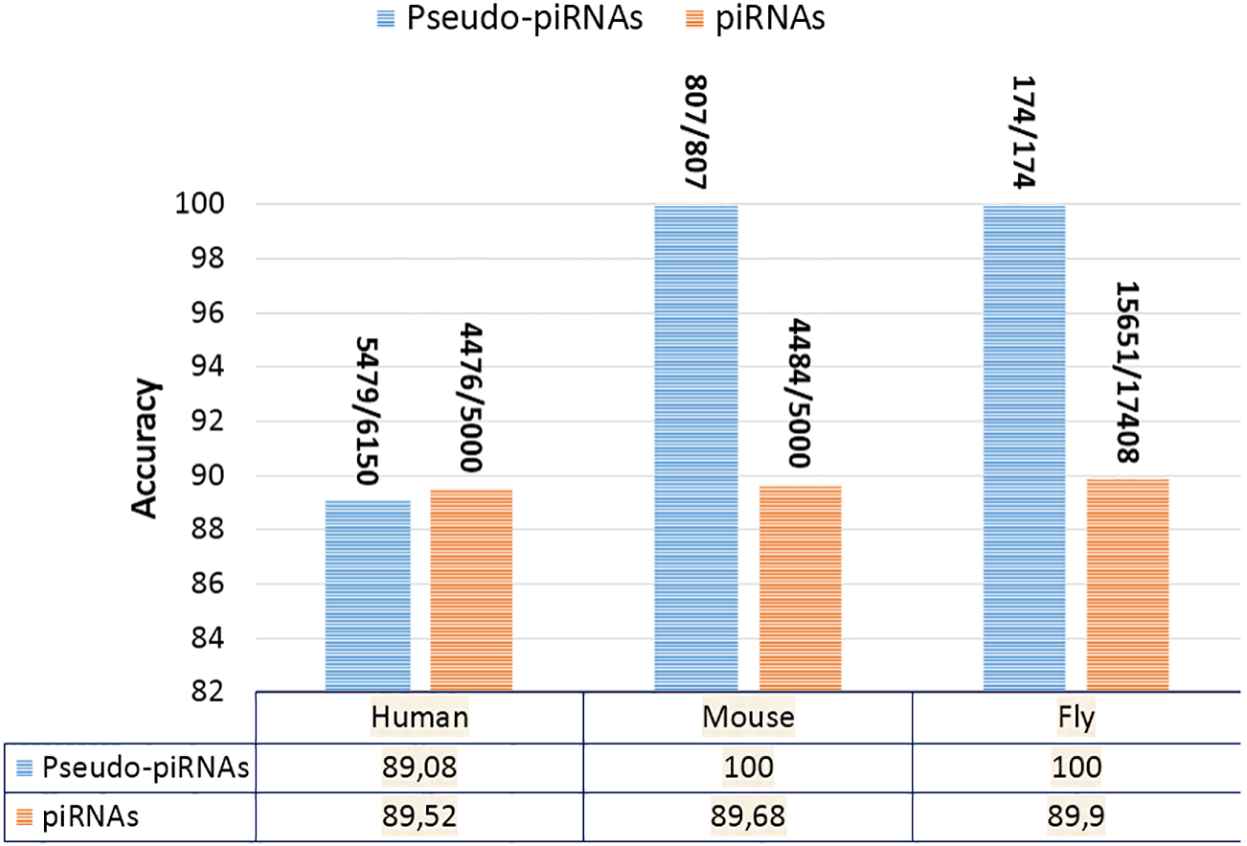
IpiRId prediction results on piRNA and pseudo-piRNA sequences across species.

Furthermore, we have assessed the predictive performances of our tool using sequences that haven’t been considered in the training process. Accordingly, we have used 5000 positive and 6150 negative sequences for human, 5000 positive and 807 negative sequences for mouse and 17408 positive and 174 negative sequences for fly. Fig 4 illustrates the predictive performance results of IpiRId over the three considered species human, mouse and fly. From this figure, it can be clearly observed that IpiRId can predict both piRNA and pseudo-piRNA sequences with high accuracy.

### Feature pertinence over species

A significant interest of our tool is that it makes it possible for biologists to measure the pertinence of a given feature regarding the considered species. Obviously, the features are often observed experimentally in one or many species, as shown in Table 1.

**Fig 5:**
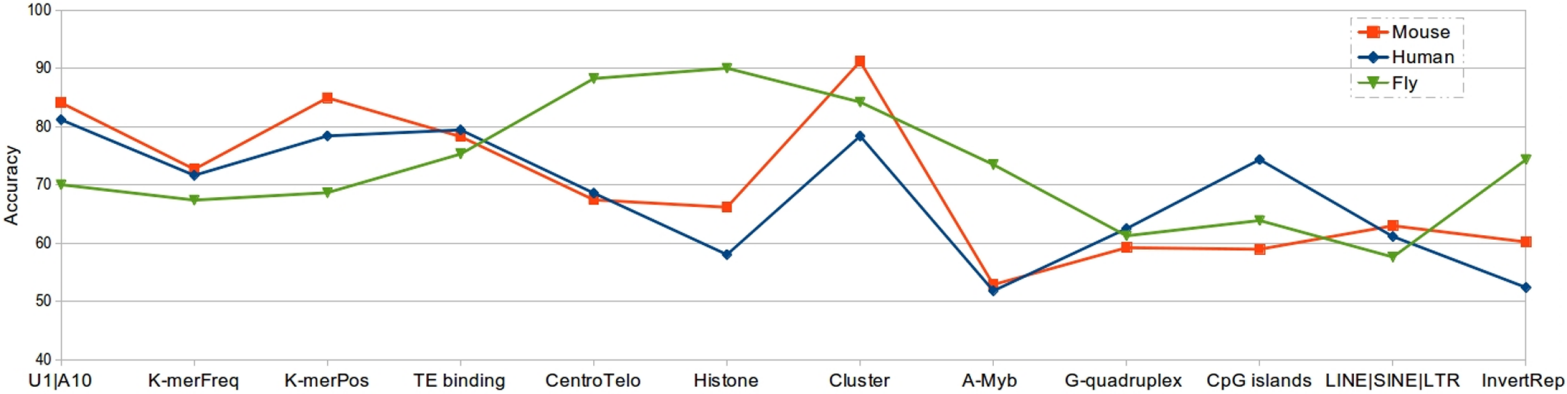
IpiRId’s features pertinence across species: Mouse, Human and Fly.

Here, we present through our computational results the pertinence of each of these features. The results shown in Fig 5 confirm that the features of first ‘U’ and tenth ‘A’, the occurrence in clusters and the binding with transposons, which were observed in several species, mammals and insects, are the ones that better characterize piRNAs in all studied species. The kernels implementing these features are indeed the ones giving the best prediction results (between 70 and 91% accuracy). Note that the results obtained by the TE binding kernel can certainly be improved by considering a larger set of transposons (for computational reasons, we considered a set of only 1 000 transposons in this study).

Also, the two k-mer-related kernels (K-mer frequencies and K-mer positions), which are not specific to piRNAs and could be used for other ncRNA, give good results, validating the new methodology proposed in this work for considering these important features.

Regarding the other kernels representing features observed in specific species, the results are different from one species to another, suggesting that these features are not conserved in all species. For instance, two features observed in fly seem to be not conserved in the other considered species: the distance to centromere and telomere regions and the histone methylation. The kernels implementing each of these two features give very good prediction results in *Drosophila*, with an accuracy around 90%, while, in human and mouse species, they give an accuracy less than 70%.

The G-quadruplex feature, observed in human and mouse (and also in rat and macaque), gives similar accuracy results, around 60%, on the three species, which shows that this feature is not very significant, even it seems not to be due to a random event. In addition, we can make the same remark on the feature of the presence of transposons upstream or downstream of the piRNA sequence, feature that was observed in mouse and marmoset species. The corresponding kernel (LINE|SINE|LTR) gives relatively same accuracy results.

Surprisingly, two features observed in mouse do not produce significant prediction results in this species: the transcription factor (A-Myb promoter) and the piRNA cluster encapsulated by an inverted repeat. The kernels implementing these two features give respectively around 50% and 60% of accuracy. The results are quite similar for human, but however, they give relatively good accuracy results, around 74%, for fly. Note that about the A-Myb promoter, the low accuracy might be because this feature characterizes a particular sub-class of piRNAs, the pachytene piRNAs.

Another remarkable result concerns the CpG islands kernel. Since the corresponding feature is related to histone methylation in mammals, we expected to get prediction results close to the ones obtained by the histone methylation kernel. But this is not the case since for human the CpG kernel gives an accuracy of 75% whereas the histone methylation kernel gives only 58% and inversely, for fly, it gives an accuracy of 63% when the histone methylation kernel gives more than 90% accuracy.

In the MKL approach, we seek for the combination of kernels that allows to obtain the best classification results. For the three species, the MKL algorithm have associated small weights to the kernels which give the worst results (for example, in the Mouse specie, the weight of the kernel “A-Myb” is 2,32 whereas the weight of the “cluster” kernel is 34,12).

To summarize, it can be observed that the different studied species share very few features. However, our method deals with this limitation and allows to get good prediction results using all these features together.

## Conclusion

A piRNA can be characterized by its sequence, and also its positions on the chromatin, positions regarding sequence and/or structural motifs that can occur at the 5’ and/or the 3’ ends, possible occurrence in clusters, and interaction with specific target sequences. We have proposed in this work an integrative approach for piRNA identification based on MKL methodology taking into account a large set of heterogeneous features, and dealing with the non-conservation of certain features between species (thus taking into account the species evolution). The MKL method allows combining heterogeneous features by tuning automatically their weights in order to improve the prediction. We did a thorough study of possible biological features that characterize piRNAs and that could be used for their prediction by computational methods. This resulted in a large number of heterogeneous features (13 features, very few of which have already been considered in computational tools), mostly related to function and transcription. Then, we categorized these features into several principal classes and implemented them in generic and modular tool, called IpiRId, that could be easily adapted for the prediction of other classes of ncRNAs. It makes it possible to test features observed for a type of ncRNA on other ones, as well as testing the validity of new features that have never been considered.

IpiRId outperforms all existing tools for piRNA prediction, giving an accuracy around 90% in human, mouse and fly species. Finally, and thanks to our MKL method and modular tool, we could measure the importance of each feature in these three species (users could also choose the most appropriate combination of features to a specific species). In brief, our study reveals that the most conserved piRNA features across species are: first Uridine, tenth Adenine, occurrence in cluster and binding with transposons.

The running time of IpiRId depends on the number of sequences used in the training step to build the prediction model as well as the number of the selected kernels. For example, the time required to predict 10 sequences in fly is around 8 seconds based on the model built using 2 200 sequences. To improve the running time and the computational performances of IpiRId, we are working on a parallel version where the the different kernels are built in parallel.

In our approach, we have used the L2 regularization which associates smooth weight values to each kernel because it gives better classification results. In future work, we will test the consequence of the use of L1 or Lp (0 < p < 1) norms, which leads to sparse weight values (this represents a sort of feature/kernel selection) on the running time and the result interpretation.

Finally, an ongoing work concerns the development of tools for the prediction of other classes of ncRNAs (miRNAs, snoRNAs, circRNAs, …), by integrating other kernels implementing specific features of each of these ncRNAs.
